# 

**Published:** 1885-01

**Authors:** 


### NOTICE.

Original communications solicited. When articles require illustration the engravings will be furnished by the publishers free of charge.

Subscription: $2.50 a year.

Subscribers will please not send us money by Postal Notes. Remit by Express, Draft, Post-office Order, or Registered Letter.

Address all letters and books, and make all drafts payable to

The Atlanta Medical and Surgical Journal,
Care Jas. P. Harrison & Co., Atlanta, Ga.

